# 非小细胞肺癌伴神经内分泌分化的研究进展

**DOI:** 10.3779/j.issn.1009-3419.2011.02.11

**Published:** 2011-02-20

**Affiliations:** 1 200438 上海，上海五角场社区卫生服务中心 Department of Respiratory Medicine, Shanghai Wujiaochang Hospital, Shanghai 200438, China; 2 200030 上海，上海交通大学附属胸科医院呼吸科 Department of Respiratory Medicine, Shanghai Chest Hospital, Shanghai Jiaotong University, Shanghai 200030, China

肺癌是最常见的恶性肿瘤，已成为人类因癌症死亡的主要原因之一。在肺癌治疗前明确肿瘤的类型十分重要，因为它和患者的个性化治疗密切相关，并能为肿瘤的流行病学及生物学研究提供基础。目前，肺癌按病理类型分型较多，而且根据某些形态学和超微结构，肺癌中还有一些具有神经内分泌形态学的肿瘤，能较好地明确肺癌的病理类型及亚型，可以对这些异质性肺癌的生物学特性的研究及开展个性化治疗提供较多的临床依据^[1]^。近年来发现，肺癌还伴有神经内分泌分化（neuroendocrine differentiation, ND），研究证实神经内分泌分化现象广泛存在于前列腺、胃肠道、肺等部位的癌中。世界卫生组织（World Health Organization, WHO）肺肿瘤组织病理学分类新增加了非小细胞肺癌（non-small cell lung cancer, NSCLC）伴神经内分泌分化的概念，由于对该类异质性肺癌研究较少，许多学者都对其生物学特性和对化疗的敏感性及对生存期的影响存在着争议，本文就神经内分泌分化标志物的选择和检测以及神经内分泌分化与NSCLC的化疗疗效、生存期的关系和相关的基因将国内外的研究进展作一综述。

## 1 肿瘤的神经内分泌分化

恶性肿瘤有着许多特征，但基本特征之一是细胞的异常分化。在肿瘤细胞向不成熟方向退行性发育的过程中，可出现原来组织或器官中的正常细胞所没有的新的分化特征或原有一些特征的丢失，这种分化不仅给肿瘤的诊断带来困难，同时也给肿瘤治疗造成了许多障碍。肿瘤组织内的ND是指肿瘤组织中部分肿瘤细胞出现ND现象，它不同于一般内分泌腺肿瘤和神经内分泌肿瘤。肿瘤组织中这部分ND的肿瘤细胞为肿瘤组织的一种伴随成分，和神经内分泌癌区别为按照WHO分类标准：肿瘤组织中一种神经内分泌标记物明确阳性，且阳性细胞数＞50%时诊断为神经内分泌癌，否则为伴神经内分泌分化。恶性肿瘤细胞向ND方向分化，但它仍具有恶性特征，即在癌病灶中的ND细胞既显示肿瘤主体细胞的结构特点，又显示胞浆内分泌颗粒。正常ND细胞具有类似神经细胞的致密圆形胞体和大量细长而有分支的树状突起。在腺癌病灶中的ND细胞突起缺乏特征性。在低分化腺癌中ND细胞呈圆形、卵圆形或不规则形，极性消失，这些细胞的胞核与周围肿瘤一样具有明显的异型性。在高分化腺癌中，ND细胞呈锥形或长条形，顶端朝向腺腔侧，有时可见其突起抵达腔面^[2, 3]^。

ND现象已经在多种非神经内分泌器官中得到证实。由于分化的神经内分泌细胞在癌组织成分中不足50%，且以单个细胞或细胞巢的形式分散存在，为癌组织的一种伴随成分，被称为癌伴神经内分泌分化，从而与神经内分泌肿瘤相区别。这些散在的神经内分泌细胞与其所在器官原发癌的类型、分期、分级和预后关系密切。许多研究证实，神经内分泌分化现象广泛存在于前列腺、胃肠道、肺等部位的肿瘤中。这些分化的神经内分泌细胞可合成、分泌各种神经肽或者胺类激素，致使血液中这些激素类物质的水平升高，有时可因激素过剩引起相应的神经内分泌症状，即副神经内分泌综合征。除甲状腺外，胃肠道、前列腺和乳腺、胰腺等组织的正常上皮中均含有神经内分泌细胞细胞。与正常神经内分泌细胞的形态特征类似，分化的神经内分泌细胞胞浆内含有多种致密核心颗粒，它们与存贮和分泌大量内分泌活性物质有关^[4]^。

## 2 肺的神经内分泌肿瘤

自1981年WHO分型公布后，对某些肺肿瘤已有了更进一步的认识，肺神经内分泌肿瘤的概念被重新界定。肺的神经内分泌肿瘤是一种独特的亚型，它拥有某些形态学、超微结构和分子学特征。在组织病理学上能识别的类型有：小细胞肺癌、大细胞神经内分泌癌、不典型类癌和类癌。在W HO新分类中将它们统称为具有神经内分泌形态的肺肿瘤。然而，这些肿瘤各有不同的流行病学、发生学、组织形态学、分子生物学特性及临床预后^[5]^。小细胞肺癌是这类肺癌中最具临床特点的，其生物学特性和对化疗的反应性与大多数无神经内分泌分化的肺癌有明显区别。从分子生物学的角度看，p53的突变率和/或突变型p53蛋白在小细胞肺癌和大细胞神经内分泌癌中的表达率为50%-80%，在典型类癌中不表达，在不典型类癌中仅有10%-20%；在3p、13q14、9p和5q22的杂合子丢失频率，从不典型类癌到大细胞神经内分泌癌和小细胞肺癌也是逐级增加，所以，这种神经内分泌肿瘤分类具有临床预后意义^[6]^。

近年来研究^[7]^发现一部分NSCLC中也具有ND特征，国内外研究发现其发生率在10%-30%。约10%-20%的鳞癌、腺癌和大细胞癌具有神经内分泌分化的特性，在腺癌中最常见，其阳性率与使用的标志物和检测技术密切相关，这一问题已引起很大的关注。与缺乏ND的NSCLC相比，这类肺癌患者对化放疗的敏感性和预后研究的尚不深入，对其生物学行为研究的也较少，故WHO并没有将其归入肺癌的神经内分泌肿瘤内，而是在1999年“肺与胸膜肿瘤组织病理学分类标准”中，新增加了非小细胞肺癌伴神经内分泌分化（non-small cell lung carcinomas with neuroendocrine differentiation, NSCLC-ND）的概念。NSCLC-ND是指在光镜下不具有神经内分泌形态特征，但免疫组化和电镜证明有ND的NSCLC。目前免疫组化标记是检测ND较好的方法^[8]^。

## 3 肺ND特异性标记物

目前公认的ND特异性标记物是嗜铬蛋白A（chromogranin A, CgA）和突触素（synaptophysin, SYN）。嗜铬蛋白是一组相对分子质量大小不等的酸性可溶性蛋白质，构成特异性致密核心颗粒的基质，可伴随ND多肽激素出细胞。CgA是其中之一，它是一种酸性可溶性蛋白，属于调节分泌蛋白家族，由439个氨基酸组成，其编码基因位于14号染色体。主要储存在肾上腺髓质和多种神经内分泌细胞的致密核心颗粒中。有研究显示CgA的表达与致密核心颗粒出现具有一致性。CgA与各种神经肽和胺类内分泌颗粒及其它神经内分泌标志物共存，是目前国际公认的特异性较强的神经内分泌标记物^[9]^。

相对于CgA来说，SYN的研究更全面。SYN是相对分子质量为3.8×10^4^的结合膜糖蛋白。已发现哺乳类神经系统突触囊泡膜蛋白至少有15种之多。分子生物学研究揭示这些蛋白质的结构、分布和功能各不相同。目前，研究的最完善的是突触素。Wiedenmann等^[10]^在大鼠、小鼠、牛和人脑突触囊泡膜上识别了又一新的分子量为38, 000 Da的必需膜蛋白，并命名它为Synaptophysin。Navone等^[11]^详细探讨和定量研究了p38在大鼠神经组织和非神经组织的分布和亚细胞定位。Südhof等^[12]^采用重组DNA生化技术阐明了p38的分子特征及其基本结构，暗示此蛋白可能构成突触囊泡特异的膜通道。不久Thomas等^[13]^对p38的四级结构及功能特性进行了研究，证实p38与通道蛋白具有同样的结构特征，表明其在神经递质释放过程中起重要作用。鉴于p38的分子结构和氨基酸序列的确证，p38的免疫细胞化学及原位杂交技术已广泛用于动物实验研究，且有部分用于人类，用来检测p38的分布以及评估突触数量和突触前终末密度，在临床方面有潜在应用价值，如神经内分泌肿瘤的诊断，肿瘤发生来源及疾病机制的探讨。突触素存在于脑、脊髓、视网膜中神经元的前突触囊中，还有肾上腺髓质中相似的囊腔中以及神经肌接头处。突触素因此成为前突触囊膜的分子标志物，并且在突触囊腔的形成以及胞吐过程中起作用。突触素SYN抗体可以标记存在肾上腺髓质颈动脉小体、皮肤、垂体、甲状腺、肺、胰腺、胃肠道粘膜、中枢神经、脊髓神经以及视网膜中的神经内分泌细胞。该抗体可以和多种神经内分泌肿瘤反应，如神经母细胞瘤、成神经细胞瘤、嗜铬细胞瘤、非嗜铬性神经节细胞瘤，还可以和上皮性的神经内分泌肿瘤发生反应，如呼吸道和胃肠道的神经内分泌肿瘤。

以往的实验有用NSE、NCAM、TNF受体，ProGPR，LEU-7等作为标记物来检测NSCLC的ND^[14]^，但其敏感性和特异性都不如CgA和SYN在NSCLC中高^[15]^，NSE标记物现在被认为是无帮助的，因为在NSCLC中，2/3 NSE染色阳性，特异性较差。检测ND的方法很多，如电镜、免疫组织化学（immunohistochemistry, IHC）测定ND相关的酶和原位杂交检测编码ND标志物的mRNA等，Slodkowska等^[16]^研究认为免疫组化是目前测定ND的标准方法，因为免疫组化在鉴定神经内分泌肿瘤上无论从形态学或从诊断到以后的治疗，在病理上都是无可取代的方法。

国内研究发现CgA阳性表达率为10%-30%，SYN阳性率为15%-39%。Sundaresan等^[17]^认为腺癌伴有ND的发生率高于鳞癌，低分化癌的ND高于高分化，CgA和SYN一般在腺癌中表达较多，CgA在不同分化程度的肿瘤中表达较接近，而SYN在低分化癌中表达显著，说明肿瘤分化程度越低，越容易形成ND，产生异质性肺癌细胞。

## 4 NSCLC-ND对患者生存期的影响

对肺癌术后生存情况的预测，目前较明确的依据是肿瘤的生物学特性^[18]^。越来越多的学者致力于对肺癌伴神经内分泌分化现象的研究，分析它们与肺癌恶性程度的关系，以指导对肺癌预后的治疗^[19, 20]^。NSCLC-ND对预后的影响在国外一直有争议。Pelosi等^[21]^对220例Ⅰ期的NSCLC研究发现，ND超过肿瘤细胞5%的患者预后较差，Ⅰ期NSCLC伴ND患者生存期较无ND的NSCLC低，且有明显差异。他们在220例Ⅰ期NSCLC患者中，用光镜和IHC检测Cg A、Syn和呼吸道相关激素，其中28例为NSCLC伴ND，包括5例腺癌和13例鳞癌患者。5年的随访所获结论为NSCLC伴ND特别是病理为腺癌且超过5%肿瘤细胞有ND特性的患者，其生存期比普通的NSCLC患者短（*P*=0.017），预后差。而Howe等^[22]^对439例NSCLC患者的研究提示无论采用手术还是化疗的NSCLC患者，他们的生存期与神经内分泌分化无关，因此Howe认为不能用现有的检测ND的免疫化学指标（包括CgA、Syn和NCAM）对NSCLC疾病进行预后评价。Nisman等^[23]^研究了67例晚期NSCLC发现治疗前CgA高表达不利于生存。ND是影响生存期的独立因素。Graziano等^[24]^检测了260例手术切除的Ⅰ期、Ⅱ期NSCLC患者的NSE、CgA、Leu-7和Syn等标志物，结果显示NSE的阳性率为70%，CgA为14%，Leu-7为8%和Syn为11%，其中61例患者有2个或2个以上ND标志物阳性。1个ND标志物阳性与2个或2个以上ND标志物阳性患者相比无病生存期无差异。这几项研究结果的不一致，可能由于各研究中化疗方案有差异，另一方面研究的NSCLC临床分期也不一致，肺癌细胞在早晚期的异质性差异也有可能使得研究结果有所差异。由于可能存在其它影响生存期的混杂因素，Gonzalez等^[25]^把病例限制在Ⅰa期和Ⅰb期的鳞癌和腺癌手术患者中，避免了亚型的混杂因素的干扰，主要研究SYN的表达，发现SYN阳性表达的患者肿瘤更具侵袭性，预后更差。Ionescu等^[26]^研究了更多的肺癌标本，得出的结果却是ND不能作为预后的判断指标，由于其文章中未列出肺癌的分期情况，其病例中还包括癌肉瘤和巨细胞癌，其结果还待多中心临床试验来明确。Gregorc等^[27]^使用ELISA和IHC的方法来检测CgA和TNF在晚期NSCLC患者血中的浓度和蛋白的表达情况，发现血清CgA和肿瘤组织中的CgA均能作为预后的预测因子。CgA血清浓度越高和蛋白表达阳性率越高，NSCLC患者预后越差。虽然以上几项研究的结果不全一致，但综合几项研究的结果，ND还是对NSCLC患者的生存期有着一定的影响。

## 5 影响ND的基因

目前对ND现象的研究大多停留在蛋白的水平，关于ND相关基因以及这些基因在ND中作用机制的研究甚少。Kazanjian等^[28]^报道hASH1与肺癌中的ND存在一定的关系。研究显示hASH1在调节肺癌ND中具有独立性作用，体外培养发现，hASH1在神经内分泌细胞和发生ND的细胞中呈阳性表达，此外还发现hASH1在具有ND的肺癌中呈特异性表达，并在调节肺癌ND中也具有很重要的作用，这些均说明hASH1与肺癌ND具有相关性。Jiang等^[29]^研究hASH1在胚胎早期的神经和肺的发育中呈短暂性表达，但是当这些细胞分化成熟、神经表型出现后hASH1的表达立即消失，并发现hASH1的表达与组织分化的程度相关，并广泛存在于低分化的腺癌组织中，这些均表明，hASH1在腺癌组织的ND中具有重要的作用。Osada等^[30]^认为虽然hASH1在肺癌的ND起着重要作用，但进一步研究发现其功能不过是作为一个转录因子来激活ND，这个蛋白通过ASH1介导脱去乙酰基来抑制DDK1、DKK3和负调节Wnt/beta-catenin和E-cadherin的信号传导。

Mountzios等^[31]^认为肿瘤抑制基因*TP53*和*RB1*的点突变和ND有关，虽然这种突变在各种肺癌的病理组织都可见，但是其突变的频率和发生的“时机”在NSCLC和有ND的小细胞肺癌中明显不同。D'Alessandro等^[32]^分析了20个新鲜的肺部的肿瘤组织，认为n-ELAV也称为Hu抗原，其基因的改变和肺部肿瘤的ND的开始以及后续的一系列侵袭变化密切相关。

## 6 展望

恶性肿瘤细胞朝神经内分泌方向的分化是肿瘤细胞在侵袭演进过程中的一种分化，还是肿瘤组织本身所具有的一种非特异性的变化，值得我们继续探讨。ND发生可能涉及到众多调控环节，但具体的调控位点仍然是未知，因此对恶性肿瘤伴ND发生发展机制的研究有重要的理论价值。在NSCLC中进一步研究ND现象，对于其生物学特性和亚型的研究，寻求预防恶性肿瘤浸润转移、防止肿瘤细胞抗药性及研制诱导分化的新的治疗方法及药物，都有着重要意义。
